# A new method for the treatment of unilateral posterior cross-bite: a three-dimensional finite element stress analysis study

**DOI:** 10.1186/s40510-018-0227-z

**Published:** 2018-08-27

**Authors:** Çağrı Ulusoy, Merve Dogan

**Affiliations:** 1 Department of Orthodontics, Faculty of Dentistry, Gazi University, Emek, Ankara, Turkey; 2 Antalya, Turkey

## Abstract

**Background:**

Stress relieving corticoto mies during the treatment of maxillary expansion are needed in adult patients.

**Methods:**

Three-dimensional (3D) finite element model was prepared, and finite element analysis was processed to evaluate the stress distributions within the skull and maxillary teeth during surgically assisted rapid maxillary expansion (SARME) treatment.

**Results:**

Expansion forces generated more stress on the corticotomy-applied part of the maxilla. The stress levels decreased dramatically above the corticotomy line.

**Conclusion:**

Asymmetric transveral maxillary expansion might be achieved from a symmetric force generating screw during SARME treatment. SARME osteotomies may concentrate the stress in the expanding maxilla and reduce the pain in other parts of the cranium.

## Background

Posterior cross-bite, which may be occurred by skeletal, dental, or functional reasons, is one of the most common craniofacial disorders in transversal direction [1]. Unilateral posterior cross-bite is a specific subtype of this disorder characterized by an arch deficiency. It may alter the mandibular growth pattern of the growing subjects and form asymmetric condylar height resulting in facial asymmetry [2].

The patient’s age, the complexity of the disorder due to sagittal and vertical maxillo-mandibular relations, and the presence of other systemic problems should be considered in the treatment planning of unilateral cross-bite [3]. Orthodontic, orthopedic, or combination of orthopedic and surgical skills could be used in increasing the transversal width of the maxilla. Surgically assisted rapid maxillary expansion (SARME) was one of the most effective methods in the treatment of unilateral cleft palate problem [4]. Paralysis of the nerves, hemorrhage, pain, deviation of the nasal septum, periodontal diseases, and relapse of the treatment were shown among the possible problems which might occur during maxillary expansion without surgical corticotomies [5].

Treatment of unilateral cross-bite was performed by either slow palatal or rapid maxillary expansion, generally resulting in an unwanted overdevelopment of the side that had normal pretreatment transversal relation with the mandibular teeth [6, 7]. Therefore, the anchorage performance of the normal side should be increased by suggesting cross elastics to the patient in order to overcome this problem [8].

Three-dimensional (3D) computer-assisted researches became popular in dental field in the past two decades [9, 10]. 3D finite element stress analysis (FESA) is a numeric method for simulating mechanical behaviors of real physical systems and considered to be a valid and reliable approach for calculating stress and displacement of dentoalveolar structures [11]. Yang et al. [12] stated that FEM could be beneficial to simulate orthodontic approaches and to compare their biomechanical effects without increasing number of patients or animals like in the clinical investigations. The aim of this study was to evaluate the effects of a new method for unilateral maxillary expansion by using 3D FESA.

## Methods

This study was conducted under the approval of Gazi University Institute of Health Sciences and study material was selected from the archive of Gazi University Faculty of Dentistry Department of Orthodontics (Approval number 2016/0493). Written informed consent of the patient was present in patients file as a regular procedure. All teeth were present in the mouth of the patient except for the third molars which might have interfered with the osteotomy lines above maxillary tuber region.

The material consisted of computerized tomography (CT) images of a skeletal class 1 adult patient with normal vertical cephalometric values and without any craniofacial anomalies except for unilateral cross bite. The CT images were requested from the patient by Department of Oral-Maxillofacial Surgery of the faculty as a routine procedure for the patients who would undergo surgical/orthognathic treatments. The CT data which consisted of 601 sections in 0.2 mm thickness was obtained by using a cone beam computed tomography (ILUMA, IMTEC Co., Hatfield, PA, USA) in 40 s with 120 kvp, 3.8 mA.

**Fig. 1:**
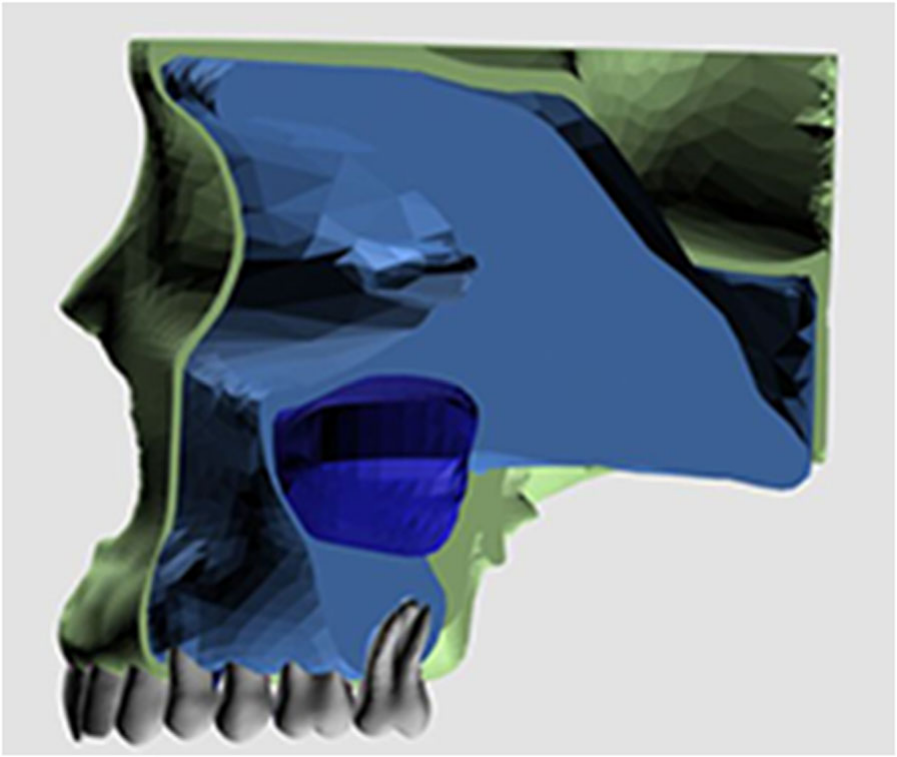
Cortical bone layer, spongious bone, maxillary sinus, and teeth from the sagittal view

3D finite element model was prepared and finite element analysis was processed with an Intel Xeon computer (CPU 3.30 GHz, 14 GB RAM, Intel Co., Santa Clara, CA, USA). Marc software (version 2005; MSC Software, Newport Beach, CA, USA) was used to construct the 3D finite element models for preprocessing and modeling. All the anatomical regions such as cortical bone shell, inner spongeous bone, maxillary sinuses, sutures, teeth, and periodontal ligaments around the teeth roots were modeled (Fig. 1).

**Fig. 2:**
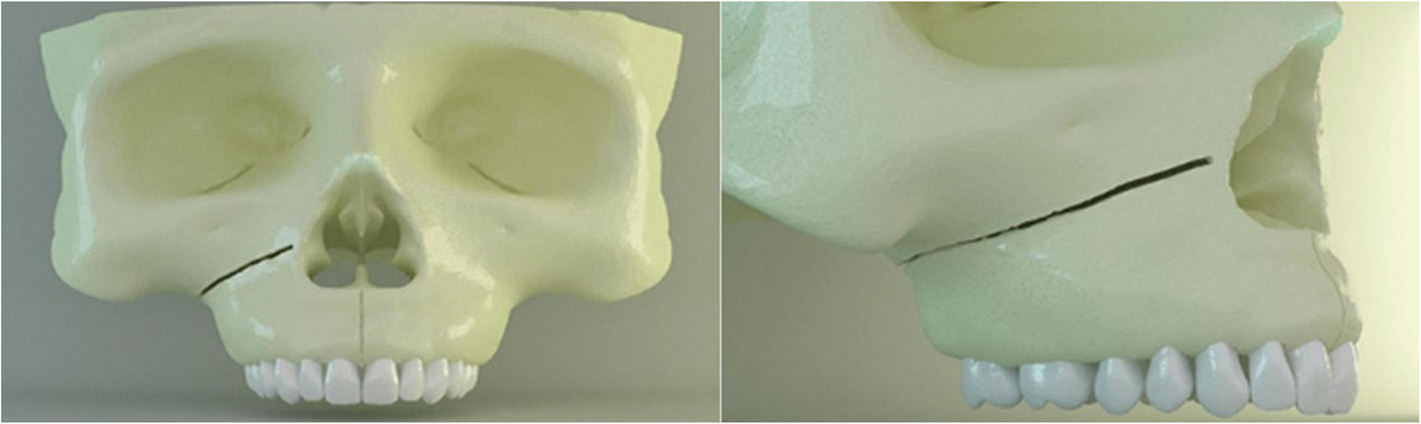
Oblique corticotomy line from frontal and lateral view. The line extends from apertura piriformis to pterygopalatinal junction lying beneath the zygomatic buttress region

The right side of the study model had an oblique corticotomy line extending from apertura piriformis to pterygopalatinal junction in the posterior, lying beneath the zygomatic buttress region (Fig. 2) [13–15]. The surgery remained in the cortical layer and did not involve the spongeous bone.

**Fig. 3:**
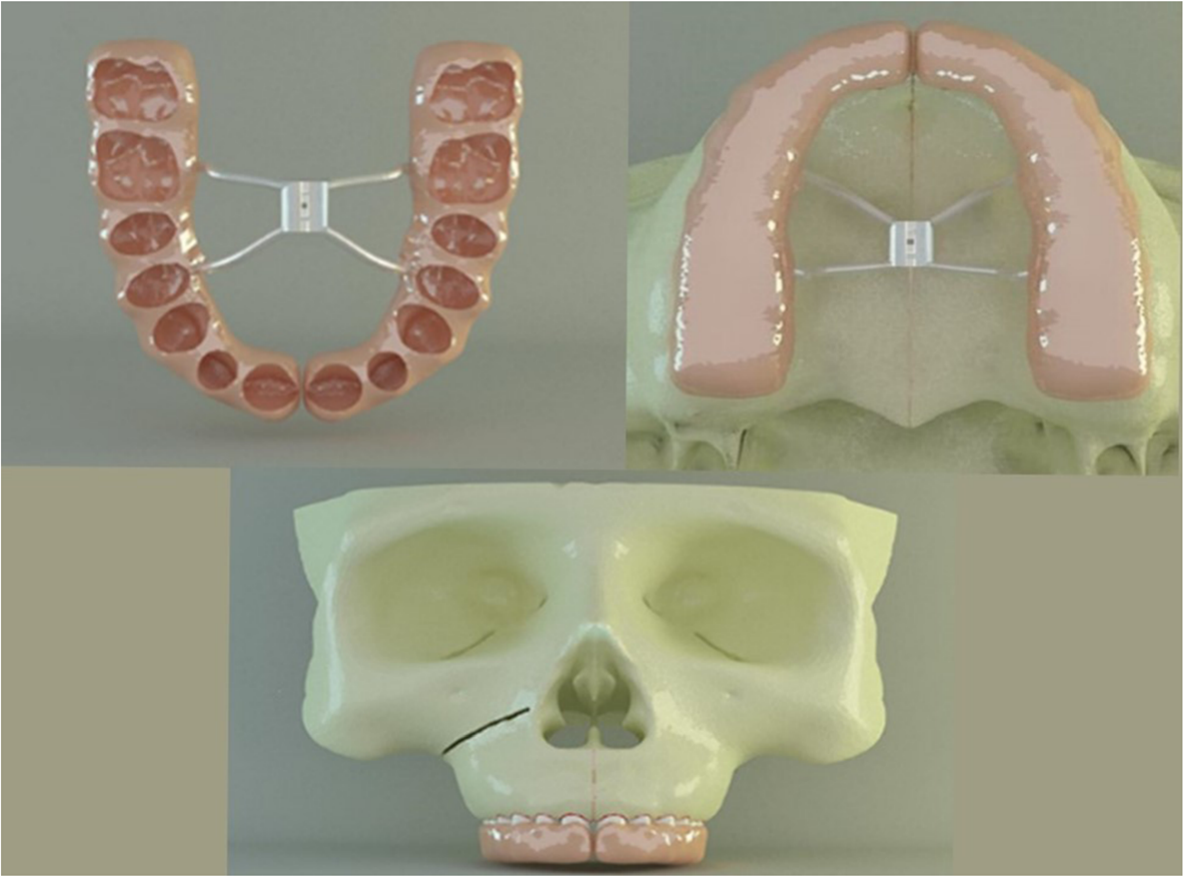
Inner view of modified full acrylic cap-splint (above left). RME was placed onto the occlusal surfaces of the maxillary teeth (above right). The gingival thirds of the teeth crowns, where the acrylic part of RME ended, were left open to achieve periodontal hygiene (below)

The rapid maxillary expansion (RME) appliance was formed on the plaster copy of the patient’s maxilla. The RME screw (Leone Orthodontics, Firenze, Italy) was set 3 mm above the mid-palatal suture. The appliance was designed as a modified full acrylic cap-splint type RME in order to eliminate the retraction force on the anterior teeth which was commonly observed during the treatment period with classic acrylic cap-splint RME’s (Fig. 3). The appliance was scanned by an optical scanner (Activity 880, Smart Optics Sensortechnik Gmbh, Bochum, Germany), and the images were based to form a 3-D RME model in computer environment by using Rhinoceros 4.0 software (Rhinoceros Inc., Seattle, USA). After the RME model was transferred to computer, it was placed onto the occlusal surfaces of the maxillary teeth. The gingival thirds of the teeth crowns, where the acrylic part ended, were left open to achieve periodontal hygiene (Fig. 3). Maxillary teeth and the inner surface of the acrylic part of the RME, which were facing each other, were bounded to simulate the bonding of the appliance to teeth in clinical conditions.

**Fig. 4:**
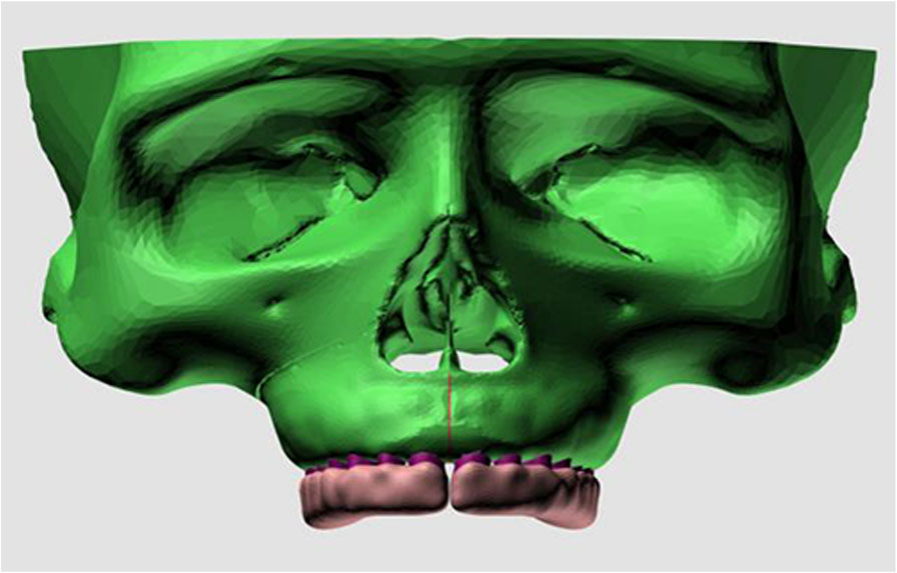
Final finite element model used in the study

Final solid meshes were constituted by hexahedral (8 noded) elements, if possible, in order to increase the reliability of the model. If a region was too small for an 8-noded element, 7-, 6-, 5-, or even 4-noded (tetrahedral) elements were formed by Marc software. The model had 183,528 nodes and 863,441 elements in total. All the bone, teeth, and RPE appliance elements were assumed to be isotropic, homogeneous, and linearly elastic (Fig. 4). The elastic properties of the materials used in this study were shown in Table 1 [15, 16].

**Table 1:** Elastic properties and Poisson ratio’s of the structures used in the study

	Elastic properties (GPa)	Poisson ratio
Cortical bone	15	0.30
Spongious bone	1.50	0.30
Teeth	19.6	0.30
Sutures	0.069	0.45
RME appiance (stainless steel)	200	0.29
Acrylic (polimethyl methacrilate)	1.80	0.35
Periodontal ligament	0.07	0.45

*GPa* gigapascal, *RME* rapid maxillary expansion

**Fig. 5:**
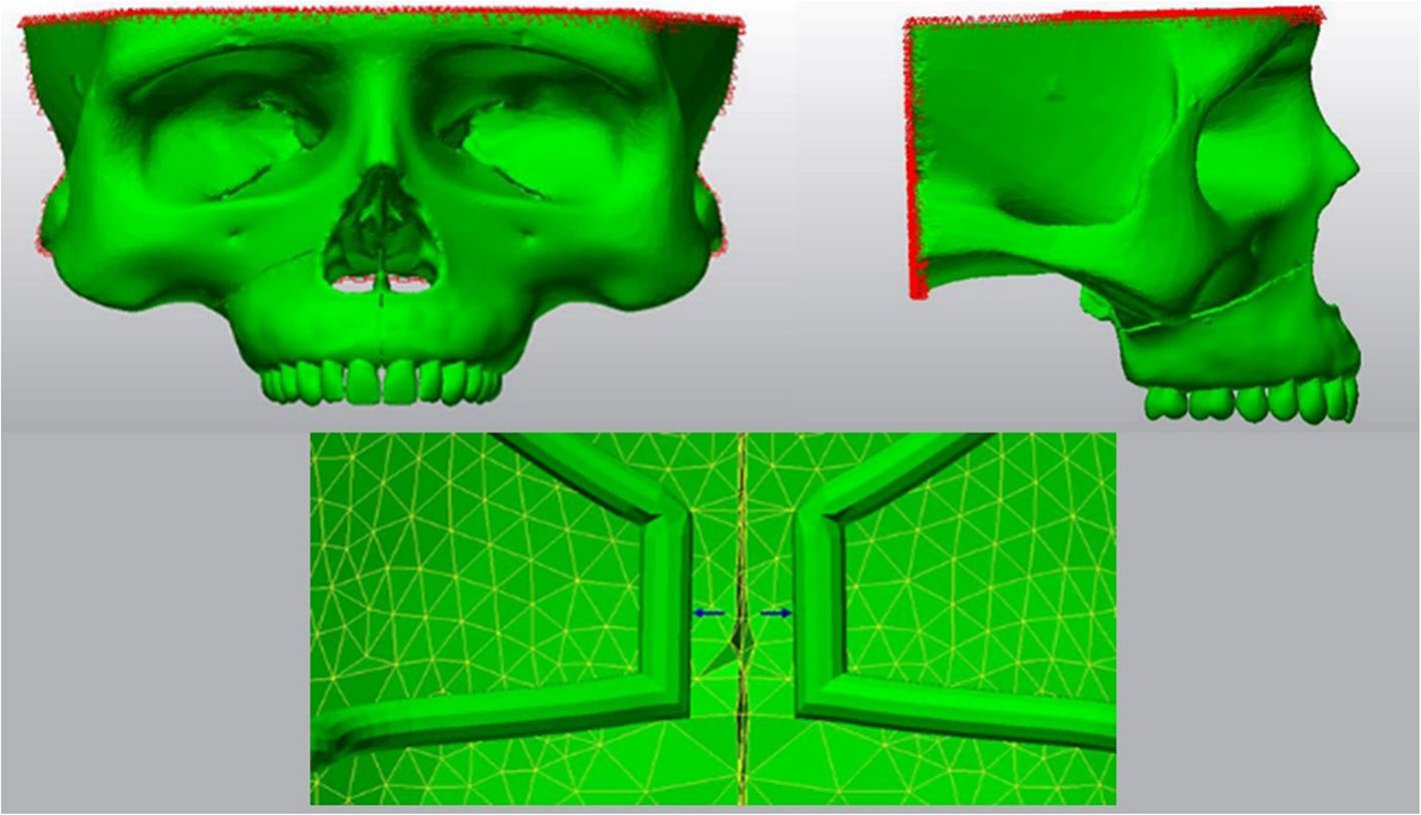
The skull model was fixed over the supraorbital region and from the posterior plane. (above left and right) The blue arrows in opposing directions show the center of force application through the mid-palatal suture (below)

Finite element stress analysis (FESA) was also performed with Marc software. The skull model was fixed with 0 degree of freedom (DOF) over the supraorbital region and from the posterior plane in order to overcome unwanted movements, shift, and rotations of the elements during force application (Fig. 5). The blue arrows in opposing directions show the center of force application through the mid-palatal suture. The magnitude of the force generated by the RME appliance was defined as 100 N [17]. This experimental setup was designed for easier evaluation and understanding of the stresses generated. Von Mises stresses generated by RME appliance were measured and evaluated by three-dimensional FESA.

## Results

**Fig. 6:**
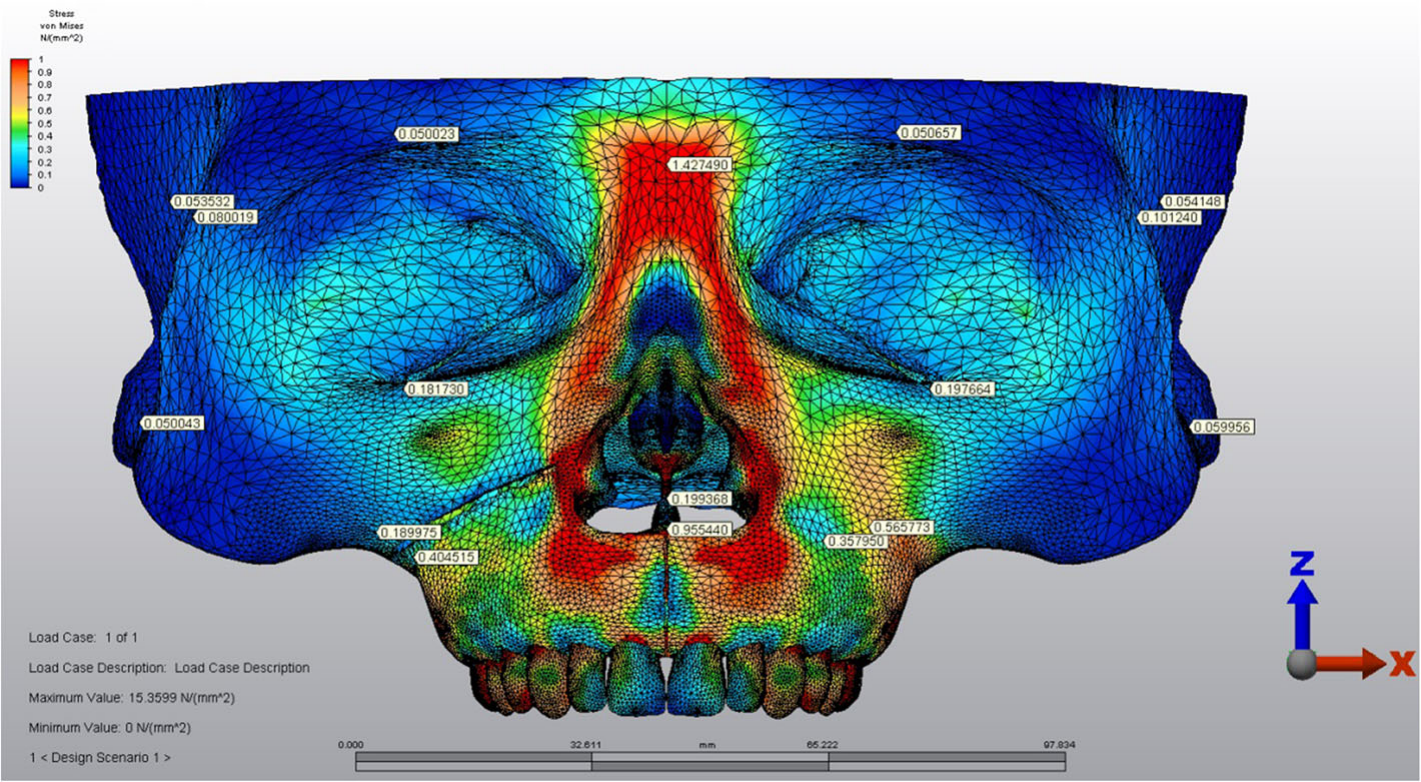
Frontal view of the stressed areas in the skull model

**Fig. 7:**
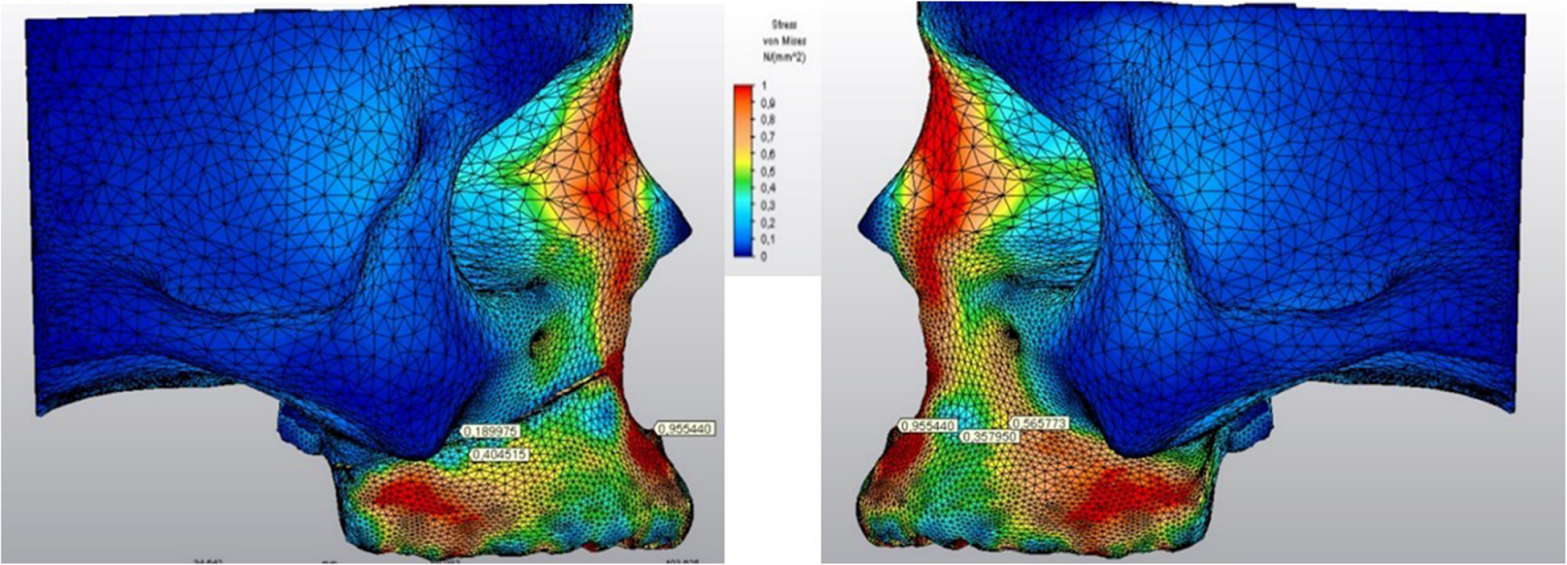
Lateral views of the stressed areas in the corticotomy applied (left) and non-corticotomy sides (right)

The stress levels in right and left sides of the skull model were generally harmonic except for the regions above and below the corticotomy line (Fig. 6). A stress magnitude of 0.40 GPa was recorded below the corticotomy line and stress was dramatically declined to 0.18 GPa above this line (Fig. 7). The stress levels in the same points of the non-corticotomy side were calculated as 0.35 and 0.56 GPa, respectively (Fig. 7).

**Fig. 8:**
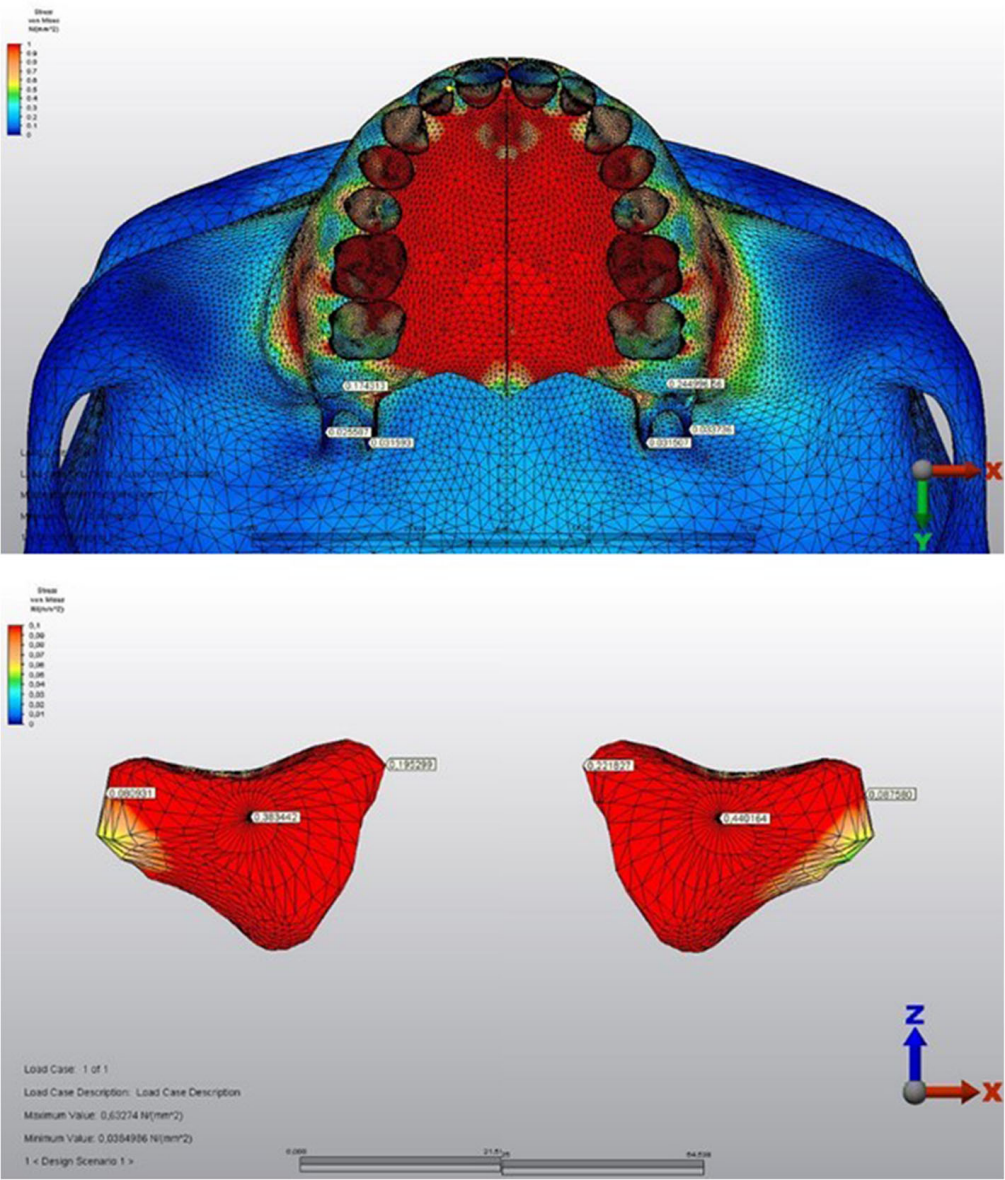
The stressed areas on the tuber, zygomatic buttress, and pterygoid processes from the occlusal plane (above). The stress levels were declined in the medio-lateral direction both on the right and left maxillary sinus walls (below). The index is descending 0.1 points from 1.0 to 0.0 as 1.0-0.9-0.8-0.7-0.6-0.5-0.4-0.3-0.2-0.1. (Red to blue)

The stressed areas on the tuber, zygomatic buttress, and pterygoid processes could be seen in Fig. 8 from the occlusal plane. The stress levels were declined in the medio-lateral direction both on the right and left maxillary sinus walls (Fig. 8). The highest Von Mises stress levels on some anatomical regions during force application could be seen in Table 2.

**Table 2:** The highest Von Mises stress levels calculated on some anatomical regions during force application

	Corticotomy applied side	Non-corticotomy side
Nasion	0.41	0.41
Anterior nasal spine	0.95	0.95
Supraorbital region	0.50	0.50
Infraorbital region	0.18	0.19
Zygomatic arch	0.08	0.10
Medial wall of maxillary sinus	0.19	0.22
Lateral wall of maxillary sinus	0.08	0.08
Anterior temporal fossa	0.05	0.05
Superior part of the zygomatic buttress	0.18	0.56
Inferior part of the zygomatic buttress	0.40	0.35
Tuber maxilla	0.17	0.24
Medial pterygoid process	0.03	0.03
Lateral pterygoid process	0.02	0.03

The values presented in the table were in GPa

**Fig. 9:**
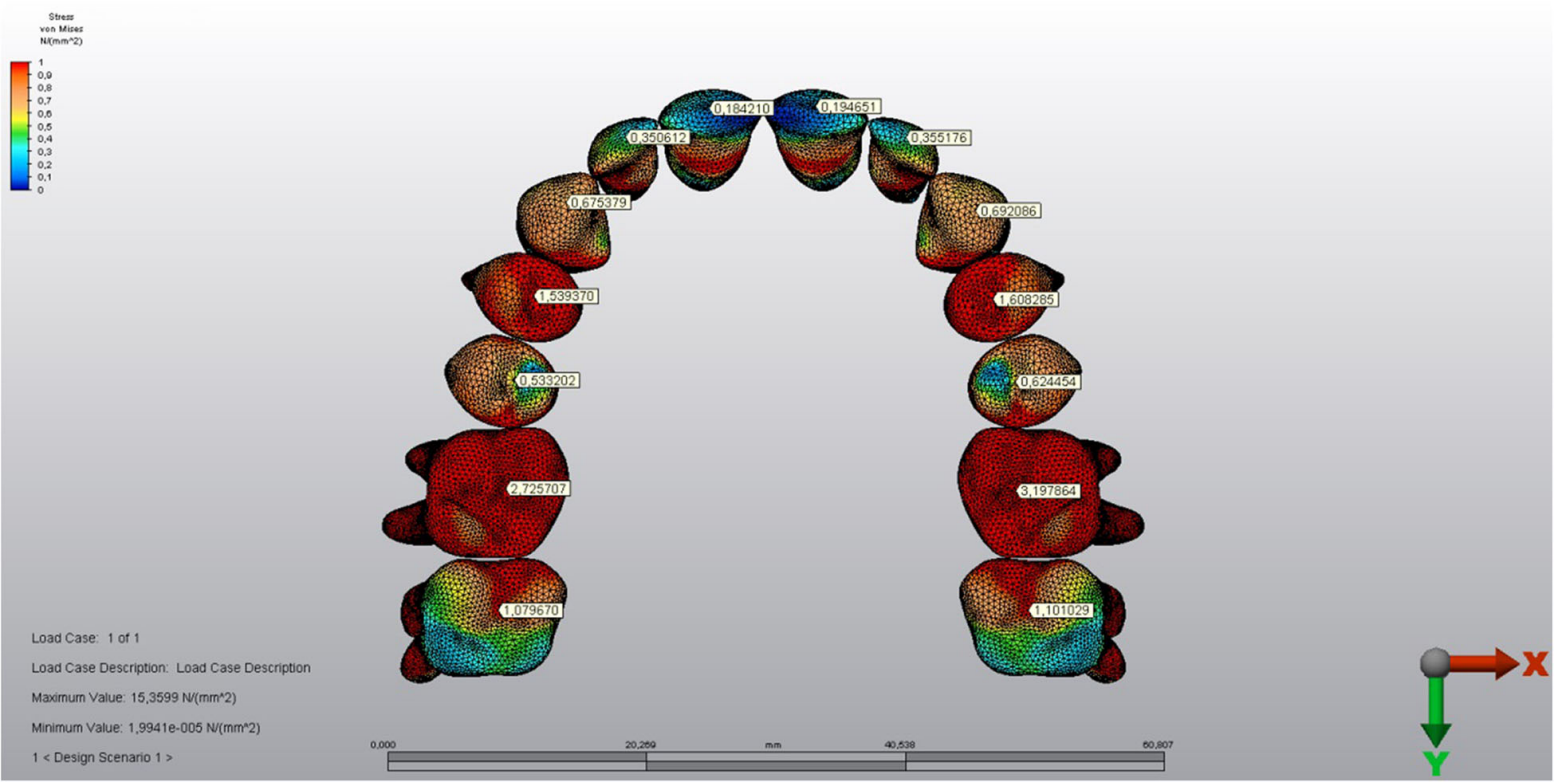
The stressed areas of the teeth crowns

**Fig. 10:**
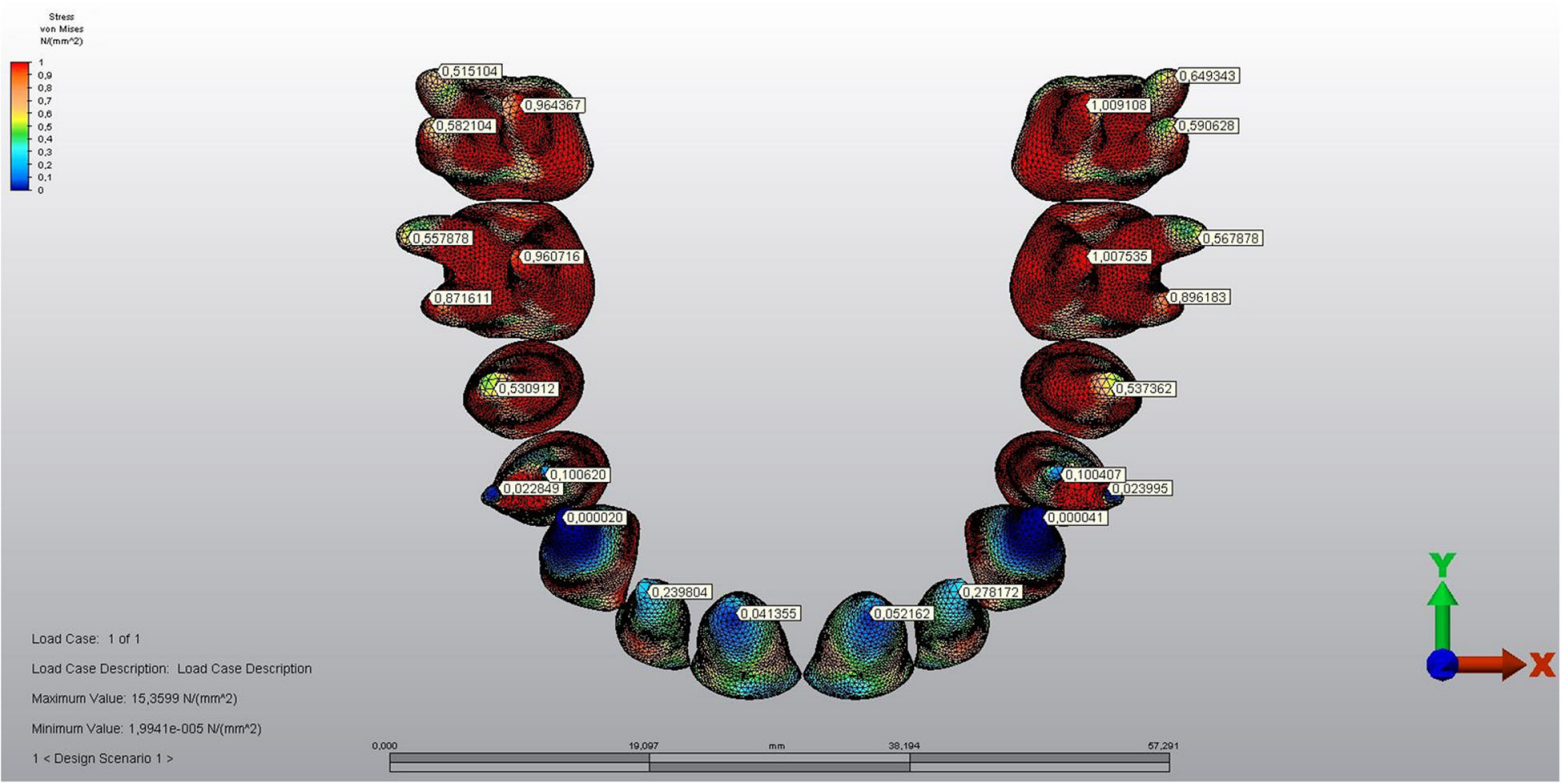
The stressed areas of the roots

The stress levels of the teeth in the corticotomy side were lower than the other side (Figs. 9 and 10). The magnitude of the stresses calculated on the teeth crowns and the roots of both sides were shown in Table 3.

**Table 3:** The highest Von Mises stress levels calculated on the crowns and the roots of the teeth

Tooth	Corticotomy applied side		Non-corticotomy side	
	Crown	Root	Crown	Root
Central incisor	0.18	0.04	0.19	0.05
Lateral incisor	0.35	0.23	0.35	0.27
Canine	0.67	0.00	0.69	0.00
First premolar	1.53	B:0.02 P:0.10	1.60	B:0.02 P:0.10
Second premolar	0.53	0.53	0.62	0.53
First molar	2.72	MB:0.87 dB:0.55 P:0.96	3.19	MB:0.89 dB:0.56 P:1.00
Second molar	1.07	MB:0.58 dB:0.51 P:0.96	1.10	MB:0.59 dB:0.65 P:1.01

The values presented in the table were in GPa. *B* buccal root, *P* palatal root, *MB*

mesiobuccal root, *DB* distobuccal root

## Discussion

In a bibliographical review about finite element modellings in dentistry, FEM was described as a prevalent and useful technique in the evaluation of stress distributions in biomechanic models [18]. During the constitution of FEM models, it was suggested that increase in the number of elements and nodes resulted in a more detailed and realistic structure [19]. In the present study, 183,528 nodes and 863,441 elements were used in model construction, which was quite satisfactory when compared with some previous three-dimensional FEM studies [20, 21].

All elements tested in the present FEM study were assumed to be isotropic, homogeneous, and linearly elastic as in the literature [14, 15]. Due to the differences between the finite element models and the actual situation owing to material properties and boundary conditions, the results of the FEM studies should be deciphered with caution. For example, the PDL is a non-linear, viscoelastic, and anisotropic material actually [12]. However, it occupies only a little volume in total skull models in FEM studies and its elastic properties are incomparably minor to dense structures as cortical bone and teeth. Therefore, insignificant assumptions like in the current example may form negligible effects on the outcomes derived from FEM analysis.

Previous studies have shown that the computed tomographic images are not reliable for generating detailed 3D models of teeth [13, 22, 23]. Dental age, tooth wear, metabolic calcium content, individual volumetric changes of dental sub-structures, and difference in hardness calibration techniques used to determine elastic properties of the tissues were shown among the factors that avoid the researchers to maintain a specific data for dentin, pulp, and enamel [24]. Therefore, a single elastic modulus and Poisson ratio data that was derived from previous FEM studies was assigned to teeth in the current study which mainly focused on the effects of unilateral corticotomy of the bone model [13, 25].

Every element in nature has an elastic limit under pressure above which the deformations stop being elastic and irreversible deformation occurs. Von Mises stress is a measure of distortion energy density at a particular point in a system which is useful in ascertaining failure in ductile materials [26]. Von Mises stress allows researchers to determine the elastic limit for any material easily; therefore, it is commonly used in computer engineering-based diagnostic experiments [9, 14, 15]. On the other hand, maximum and minimum principle stress values, defining the highest tension and highest compression, respectively, can be obtained by suitably rotating an element with no shear stress [26]. In other words, the principle stress is the normal stress that an element will ever see under specified applied loads, like hammering a nail in one direction without causing shear forces. Principle stress could be the right criterion for the FEM studies focusing on mini-screw anchorage and orthodontic tooth movement. Based on the concerns mentioned above, von Mises criterion was chosen in the present study to investigate multi-directional 3D realistic stress occurred during corticotomy applied maxillary expansion.

Rapid maxillary expansion was pointed out to be among the most promising methods in the treatment of transversal maxillary deficiency [27–29]. It was stated that the main resistance regions to the midpalatal suture opening during maxillary expansion both in clefted and healthy subjects were zygomatic buttress areas in the lateral sides and the pterygoid junctions in the posterior [29, 30]. Therefore, some relieving corticotomies should be applied over these surrounding tissues for decreasing pain during maxillary expansion in adult patients [30].

Two corticotomies, which were performed vertically between the central incisors to the anterior nasal spine and horizontally from aperture piriformis to tuber maxilla lying under the zygomatic buttress, separated the side with transversal deficiency from the maxillary body in the present study. The corticotomy in the pterygoid junction were also added to decrease the unwanted stress on this region during RME application. Therefore, the stress relieving corticotomies recommended in the literature were simulated in the present study [15, 30, 31].

The results of the current study expressed that stress values above the corticotomy line were decreased creating a more stressed area below this line. In other words, the force generated by the RME remained in the corticotomy applied maxillary part. The stress levels of the non-corticotomy side were lower below the zygomatic buttress and higher above this region. Therefore, the over-stressed maxillary half splitted by the corticotomy could be moved easily in the transverse direction whereas the other part resisted to the RME force. Our findings were harmonious with a previous study which pointed out stress changes depending on the extent of surgical approaches [13].

Similar to the findings above, the stress levels calculated on the crowns and roots of the teeth on the corticotomy side were lower when compared with the teeth of the other side. The stress values decrease from crowns to the roots on both sides, which might be interpreted as buccal tipping of the posterior teeth well matching with the classic literature [1, 32].

## Conclusions

It could be concluded that asymmetric transveral maxillary expansion might be achieved from a symmetric force generating RME screw during SARME treatment based on the results of our study. Single-sided corticotomy-based SARME may be an alternative way of treatment in unilateral posterior cross-bite. As all the findings of in vitro studies, these results should be carefully carried out in in vivo conditions.
